# Assessment of the National Disease Surveillance System in The Gambia, January 2025: best practices and lessons learned

**DOI:** 10.11604/pamj.2025.51.91.47687

**Published:** 2025-08-11

**Authors:** Violet Mathenge, Amadou Jallow, Ifeanyi Livinus Udenweze, Ebrima Jallow, Balla Jatta, Bakary Sanneh, Sheriffo Darboe, Abou Kebbeh, Musa Camara, Ebou Saine, Mary Bobb, Momodou Kalisa, Amoro Jarju, Yankuba Samateh, Mustapha Jagne, Francis Mendy, Kebba Jobarteh, Sainey Sanneh, Nfally Mballow, Lamin Saidyfaye, Lamin Manneh, Lamin Fofana, Momodou Barrow, Nuha Fofana, Alieu Sowe, Momodou Nyassi, Charles Okot Lukoya, Jane Maina, Nathan Bakyaita

**Affiliations:** 1 World Health Organization, The Gambia Country Office, Banjul, The Gambia,; 2 Epidemiology and Disease Control Programme, Ministry of Health, Banjul, The Gambia,; 3 National Public Health Laboratories, Banjul, The Gambia,; 4 Expanded Programme on Immunization, Ministry of Health, Banjul, The Gambia,; 5 Directorate of Planning and Information, Ministry of Health, Banjul, The Gambia,; 6 National Eye Health Programme, Ministry of Health, Banjul, The Gambia,; 7 Directorate of Health Research, Ministry of Health, Banjul, The Gambia,; 8 Directorate of Public Health Services, Ministry of Health, Banjul, The Gambia,; 9 Directorate of Health Services, Ministry of Health, Banjul, The Gambia,; 10 World Health Organization, Regional Office for Africa, Brazzaville, Congo

**Keywords:** Surveillance, IDSR, preparedness, response, The Gambia

## Abstract

**Introduction:**

the global trend of emerging and re-emerging diseases has highlighted surveillance as a fundamental pillar in ensuring global health security. The Gambia undertook a surveillance system assessment as part of a structured approach for surveillance system strengthening. We describe the best practices, key gaps, and critically analyse the system’s capacity to collect complete, timely, and accurate data and the use of data for meaningful decision making.

**Methods:**

we conducted a retrospective cross-sectional study to assess both core and supporting surveillance functions. A total of 74 public and private health facilities, comprising 16 hospitals and 58 primary health care facilities from rural and urban settings, were randomly sampled from a sampling frame of 195 facilities. Data were collected by MoH staff from both central and regional levels with support from officers from the WHO Country Office. Frequency distribution tables were prepared, and proportions were calculated, stratified by region and health facility levels.

**Results:**

national surveillance guidelines were available in 66% of the health facilities. Standard case definitions for priority diseases were present in 91% of the health facilities. More than half (66%) of the health facilities could handle specimens until shipment. Overall, 20%, 32% and 42% of the health facilities analysed data by person, place, and time, respectively. All regions had demonstrated the capacity to transport samples to a higher-level laboratory. At the central level, there was no budget line for surveillance or epidemic response.

**Conclusion:**

investments in emergency preparedness and response mechanisms, building workforce capacity, and strengthening sample collection and transportation were evident. However, gaps remain in data analysis, implementation of eIDSR, and preparedness planning. Improving coordination, securing sustainable funding mechanisms, and implementation of EBS and CBS present key opportunities for system improvement.

## Introduction

In recent years, the global trend of emerging and re-emerging diseases has highlighted the prominence of surveillance as a fundamental pillar in ensuring global health security [1]. Although the need for effective surveillance systems has long been recognized, experiences from various outbreaks, including COVID-19, have underscored the importance of investing in robust surveillance systems in the African region [2-4]. Robust surveillance systems help countries to predict and promptly detect affected and at-risk areas and/or population groups, thereby ensuring early warning and effective prompt response. Disease surveillance data serves as the basis for the detection of potential outbreaks to prevent public health emergencies and enables countries to monitor changing disease patterns. Notably, strong surveillance systems enable countries to design effective health interventions and to evaluate the impact of their control programmes [5].

A strong surveillance system effectively performs core functions-such as case detection, confirmation, reporting, analysis, and timely response-and is supported by enabling factors like supervision, training, adequate resources, and clear communication. To strengthen such systems, the World Health Organization (WHO) Africa Regional Office (AFRO) in collaboration with partners, developed the Integrated Disease Surveillance and Response (IDSR) strategy in 1998 as a strategy for enhancing countries´ ability to detect and effectively respond to priority diseases, conditions, and events [6,7]. The Gambia adopted the IDSR strategy in 2003 and subsequently adapted successive editions, including the latest third edition in 2022. Intending to further strengthen country preparedness and response capacities, The Gambia is among several African countries as of January 2025, rolling out the WHO Emergency Preparedness and Response (EPR) flagship initiatives aimed at strengthening countries´ capacity to prepare, promptly detect, respond to, and recover from the negative effects of health emergencies [8]. One of the programmes, Transforming African Surveillance Systems (TASS), is specifically aimed at re-imagining IDSR to enable prompt detection of disease outbreaks by generating better data and better analytics for decision making [9].

As part of a structured approach to strengthening its surveillance system, the Gambian Ministry of Health (MoH), with support from the WHO, undertook a comprehensive assessment of its National Disease Surveillance System to identify strengths, gaps, and opportunities for improvement. This was a collaborative multi-level process engaging staff from various programmes, directorates, and healthcare workers from selected health facilities across the three tiers of The Gambia´s health system. This paper documents this process and its findings, with a view to illustrating the value of conducting a comprehensive national IDSR disease surveillance system assessment. We discuss its utility in providing an in-depth understanding of the status of the existing system, highlighting surveillance gaps, critically analysing the system´s capacity to collect complete, timely, and accurate data, and data use for decision making. In so doing, the bottlenecks that impede the effectiveness and efficiency of the system are highlighted, thereby triggering evidence-based action plans for system improvement.

## Methods

**Study setting:** The Gambia, situated along the West African coast, spans approximately 400 km inland and has a population density of 227 persons per square kilometer. The country covers a total land area of 10,689 square kilometres. Based on the 2024 Population and Housing Census, the total population is estimated to be 2.42 million, with an annual growth rate of 2.5% - females constituting 51% of the total population [10]. The healthcare system in The Gambia operates through a three-tier structure. At the national level, the MoH is responsible for governance, policy development, regulatory oversight, partnerships, collaboration, and resource mobilisation. The regional level comprises seven Regional Health Directorates (RHD) tasked with implementing MoH policies and managing healthcare delivery. The RHDs oversee the provision of health care delivery and provide stewardship for primary (village health services, community clinics, and minor health centres) and secondary levels (major health centres) of care in the peripheral health facilities, constituting part of the service level of the health system. Tertiary health care centres, also part of the service delivery level, consist of the hospitals (district and general), including one teaching hospital, which is the highest level of the referral system. Some of the hospitals are semiautonomous but are required to report to their respective RHDs.

**Figure 1:**
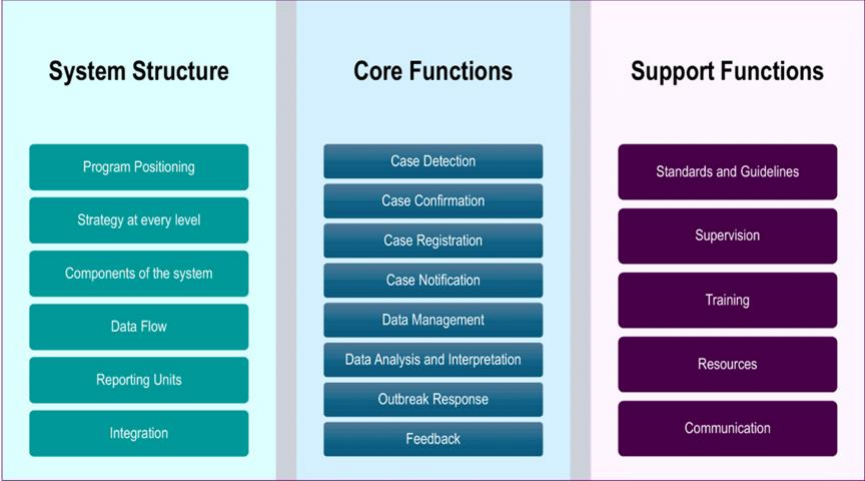
scope of the surveillance system assessment in The Gambia

**Figure 2:**
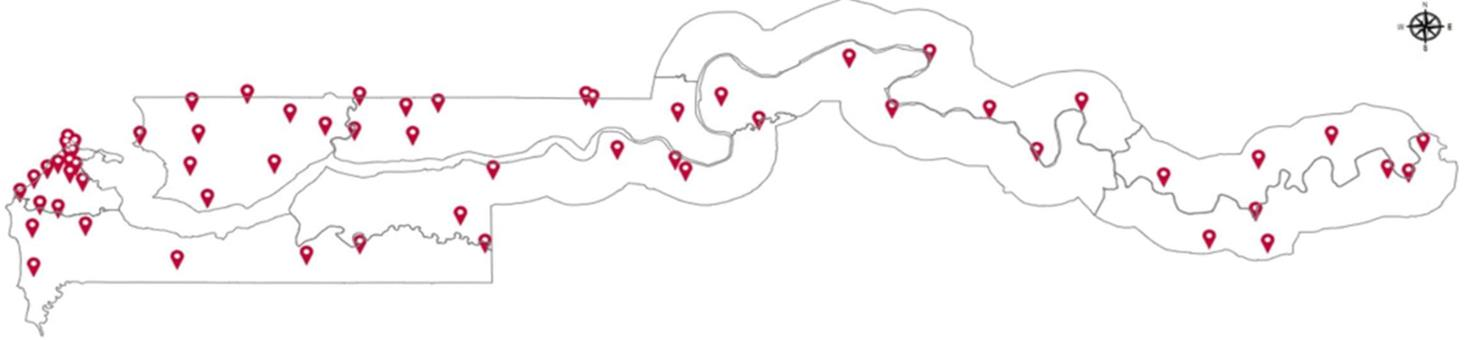
geographical distribution of national surveillance system study sites

**Study design and sampling:** a retrospective cross-sectional study was conducted to assess key areas of the surveillance system, specifically: its structure, the components and processes involved, and its capacity, based on both core surveillance functions and supporting surveillance functions outlined in subsequent sections (Figure 1). Stratified random and purposive sampling were employed, treating each of the seven regions as a distinct stratum and purposively sampling all Regional Health Management Teams (RHMT) and hospitals. The remaining health facilities were then chosen using simple random sampling from an existing sampling frame of 195 health facilities, ensuring representation across all strata. In total, 74 public and private health facilities were included in the sample - a number selected to capture a diverse mix of facility types, geographic settings, and administrative regions while remaining feasible, given logistical and resource constraints. This entailed 16 hospitals and 58 primary health care facilities, of which 58 were public, 13 private, and 3 service clinics, from rural and urban settings (Figure 2 and Table 1). Facilities in operation for less than one year were excluded from the study to ensure the inclusion of established facilities with sufficient operational history for meaningful assessment. At the national level, 13 programs and 3 directorates were engaged in the assessment.

**Table 1:** distribution of sampled facilities by region and facility type

Region	Community Clinics	Minor H. Centre	Major H. Centre	Hospital	Total
Central River	2	4	1	1	8
Lower River	3	3	1	1	8
North Bank East	3	3	1	1	8
North Bank West	7	2	1	1	11
Upper River	0	7	1	2	10
Western 1	1	6	3	8	18
Western 2	3	5	1	2	11
**Total**	19	30	9	16	74

**Assessment scope:** core surveillance functions-including detection, registration, confirmation, reporting, analysis and interpretation, response, and feedback-were assessed based on their operationalization as outlined in the Integrated Disease Surveillance and Response (IDSR) Technical Guidelines. Similarly, support functions such as setting standards (e.g., case definitions, SOPs, and guidelines), training, supervision, communication, and resources were evaluated in alignment with the same guidelines [6].

### Data collection

**Assessment tools:** an assessment questionnaire was developed based on the IDSR strategy with reference to the WHO protocol for the Evaluation of Epidemiological Surveillance Systems as well as the WHO guide for monitoring and evaluating communicable disease surveillance and response systems [11,12]. It was examined and modified accordingly to local contexts and needs and included open- and closed-ended questions to collect information on core surveillance and support functions, with separate sections for the central, regional, and health facility levels and the laboratory. The adapted questionnaire was then digitized and deployed to KoboCollect for data collection.

**Assessment teams:** the data collection was conducted by MoH team members from the central and regional levels, comprising epidemiologists, surveillance officers, public health specialists, and laboratory experts, drawn from various programs with support from officers from the WHO Country Office.

**Data management and statistical analyses:** data were downloaded from the KoboCollect server onto an MS Excel spreadsheet for data cleaning. Cleaned data were exported to STATA version 15.0 (College Station, Texas 77845 USA), where descriptive analysis was conducted. Frequency distribution tables were prepared, and proportions were calculated and stratified as appropriate.

**Ethical considerations:** this assessment was considered a routine public health program evaluation. Therefore, it required no ethical clearance. No personal identifiers were collected during the study. However, to maintain the anonymity of health facilities, the results were aggregated. The map of health facilities in the manuscript only depicts the locations of health facilities sampled for the assessment, ensuring confidentiality.

## Results

**System structure:** The Gambian Public Health Act of 1990 provides the legal framework empowering the Ministry of Health to monitor and evaluate the population's health status through public health surveillance [13]. The Epidemiology and Disease Control programme (EDC) under the Directorate of Health Services (DHS) of the MoH is responsible for the overall coordination of public health surveillance and managing events of public health importance in close collaboration with the Expanded Program on Immunization (EPI), disease-specific programmes, and other relevant stakeholders. Vaccine-preventable disease (VPD) surveillance is coordinated by the surveillance team of the EPI in collaboration with the EDC and the National Public Health Laboratory (NPHL).

The Directorate of Public Health Services (DPHS) is mandated to coordinate the implementation of the International Health Regulations (IHR) 2005 and to strengthen disease surveillance systems at points of entry. It monitors public health threats, facilitates early detection and rapid response, ensuring that public health measures are aligned with global health standards. The regional level is the hub and focus for integrating surveillance functions. Surveillance focal points at national, regional, and district levels coordinate surveillance activities at their respective levels. District surveillance officers are present in all districts. There is, however, no health services governance administrative structure at the district level. Therefore, they are primarily assigned roles focused on data collection and reporting from health facilities within their respective districts. At the time of the assessment, Indicator-Based Surveillance (IBS) was being implemented across all assessed health facilities. Event-Based Surveillance (EBS) and Community-Based Surveillance (CBS) had not yet been operationalized, though stakeholders in some regions reported initial awareness and preparatory activities.

**Core surveillance functions:** Table 2 summarizes the performance of the core surveillance functions of The Gambia´s IDSR system at both the health facility (public and private) and regional levels.

**Table 2:** performance of the IDSR core surveillance functions at health facility and regional levels, The Gambia

Core activity	Primary health care facilities		Hospitals			
	Public; (n =48)	Private; (n =10)	Public; (n =13)	Private; (n =3)	Facility; total (n =74)	Regional; total (n =7)
**General**	n (%)	n (%)	n (%)	n (%)	n (%)	n (%)
Availability of national surveillance guidelines	37 (77)	6 (66)	9 (69)	0 (0)	49 (66)	6 (86)
**Case detection and registration**						
Availability of standard case definitions for each priority disease	43 (90)	9 (90)	13 (100)	2 (67)	67 (91)	N/A
Availability of outpatient clinical register	46 (96)	8 (80)	12 (92)	2 (67)	68 (92)	N/A
**Case confirmation**						
Capacity to handle specimens until shipment	29 (62)	6 (60)	12 (92)	1(33)	48 (66)	N/A
Presence of transport media for stool at the facility	22 (47)	3 (30)	6 (50)	0	31 (43)	N/A
The capacity to transport specimens to a higher level						7(100)
Availability of guidelines for specimen collection, handling, and transportation						2(29)
**Data reporting**						
Availability of an adequate supply of surveillance forms in the past 6 months	32(67)	6(60)	8(62)	2(67)	48 (65)	5(71)
**Data analysis**						
Analysis and presentation of data by a person	10 (21)	0	5 (39)	0	15 (20)	4 (57)
Analysis and presentation of data by place	18 (38)	0	5 (42)	0	23 (32)	3 (43)
Analysis and presentation of data over time	21 (44)	1 (10)	9 (69)	0	31 (42)	5 (71)
Trend analysis	12 (25)	0	5 (39)	0	17 (23)	4 (57)
**Epidemic preparedness and response**						
Availability of an epidemic preparedness and response plan	N/A	N/A	N/A	N/A	N/A	0
Availability of budget line for epidemic response	N/A	N/A	N/A	N/A	N/A	0
Have a rapid response team for epidemics	N/A	N/A	N/A	N/A	N/A	7(100)
Have a Public Health Emergency Management Committee	N/A	N/A	N/A	N/A	N/A	5(71)
Knowledge of action thresholds for the country’s priority diseases	25 (52)	1 (10)	5 (39)	1 (33)	32 (43)	4 (57)
Standard case management protocols for epidemic-prone diseases	36 (75)	3 (30)	1 (77)	2 (67)	46 (75)	N/A
Availability of emergency stocks of drugs/supplies in the past 1 year	N/A	N/A	N/A	N/A	N/A	2 (29)
Experienced a shortage of drugs/supplies during the most recent outbreak	N/A	N/A	N/A	N/A	N/A	4 (57)

**Case detection and registration:** standard case definitions (SCD) and action thresholds for all priority diseases were developed and included as annexes in the third edition IDSR Technical Guidelines. On assessment, posters containing SCDs were present in 90% of the health facilities but were absent in one-third (33%) of private facilities. The majority (92%) of health facilities had paper-based clinical registers for recording client information, while the remaining facilities utilized digital registration systems or alternative methods for client registration.

**Case confirmation:** except for community clinics, 65% and 78% of health facilities had demonstrated capacity to collect samples of sputum and blood, respectively. In contrast, only 12% could collect cerebrospinal fluid (CSF) samples, nearly all of them (89%) being hospitals. Stool transport media were observed in less than half (42%) of the facilities visited, with none seen in the private facilities. All regions had demonstrated the capacity to transport samples to a higher-level laboratory facilitated by the Sample Referral Network (SRN) (Table 3). The availability of specimen management guidelines was assessed at the regional level. Only two of the seven regions reported having such guidelines; availability at the facility level across all regions was not assessed.

**Table 3:** strengths and weaknesses of The Gambia’s surveillance system

Core function	Strengths	Weaknesses
**Case detection and registration**	Syndromic Sentinel surveillance system for arboviruses	Gaps in the standard case definition application, particularly in private health facilities
	Availability of standard case definitions and action thresholds	Limited health care worker knowledge and monitoring of action thresholds
	AEFI Surveillance is integrated into VPD surveillance	Community-based surveillance and event-based surveillance have not been rolled out
**Case confirmation**	Trained HR to analyze priority disease pathogens	Lack of laboratory integration systems between public and private laboratories
	Developed SOPs for specimen collection and analysis	Intermittent stockouts of lab reagents and consumables for sample analysis
	Availability of SRN and African Laboratory Information Systems	Rigid SRN schedule: no provision for urgent, immediately reportable diseases
**Data reporting**	Use of DHIS2 in all regions and some Health Facilities	Lack of a central system for case-based data linking lab data with epidemiological data, and suboptimal utilization of DHIS2
	Case-based reporting for selected priority diseases, conditions, and events	Parallel reporting, data flow inefficiencies, and non-compliance with reporting requirements, particularly among private healthcare facilities.
	Timeliness and completeness > 80% at the national level	Predominantly paper-based reporting at the facility level, limiting real-time data access, timely decision making, and response
**Data analysis**	Capacity for basic descriptive analysis is present at all levels	Limited data analysis in practice at all levels, hindering timely detection and response, and suboptimal use of data for decision making
	Availability of harmonised denominators provided by GBoS	Limited capacity for advanced data analysis, such as modelling at the national level
**Epidemic preparedness and response**	Skilled RRTs at the national and regional level, 72 responders completed the WHO EPR SURGE training	Lack of an up-to-date country risk profile and multihazard plan
	National and subnational PHEOCs present	PHEOCs are not fully functional and PHEOC legal framework is not endorsed
	Standard case management protocols are available for epidemic-prone diseases	Lack of dedicated budget line for emergency preparedness and response

SRN: sample referral network; DHIS 2: district health information system version 2; GBoS: Gambia Bureau of Statistics; RRT: rapid response teams; PHEOC: Public Health Emergency Operation Centre; WHO: World Health Organization; EPR: emergency preparedness and response; SURGE: strengthening and utilizing response groups for emergencies

**Data reporting:** the country relied on paper-based tools and the District Health Information System version 2 (DHIS2) for reporting, with facilities either submitting paper-based aggregates to the regional level for entry into DHIS2 or directly entering them in the DHIS2. Less than half (47%) of health facilities used DHIS2 to report their data. In the absence of a digital system, staff reported either using text messages, WhatsApp, or physical delivery of weekly and monthly reports. In contrast, all the regions used DHIS2 for weekly and monthly reporting to the Health Management Information System (HMIS) unit. A DHIS2 tracker was in use for COVID-19, Malaria (in regions targeted for elimination), and Tuberculosis, making case-based data available for these conditions. Aggregated numbers of Adverse Events Following Immunization (AEFIs) were also recorded on the DHIS2 platform. However, serious AEFIs were investigated and reported individually using a dedicated Open Data Kit (ODK) form developed by the AFRO VPD team. The surveillance team of the EPI program maintained case-based national surveillance databases for polio, measles, yellow fever, neonatal tetanus, and meningitis. Sixty-five percent (65%) of health facilities and 71% of regions reported maintaining an adequate and consistent supply of surveillance forms during the previous six months.

**Data analysis:** overall, the capacity for basic descriptive analysis was present at the central, regional, and facility levels. Harmonized denominators provided by the Gambia Bureau of Statistics (GBoS) were available and were in use across programs. However, on assessment, limited analysis was conducted at the facility level, with the majority lacking any visible recent graphs, maps, or charts. Less than a quarter of all the facilities conducted trend analysis, while only 1 (8%) of the private facilities conducted any form of data analysis. Only 58% of regions performed descriptive analysis, while the central level mainly performed and presented it during outbreaks or public health events. Notably, the EPI regularly calculated surveillance indicators for VPDs, particularly polio, measles, and yellow fever. Automated scripts were in place to generate surveillance indicators in tables, charts, and maps, with outputs increasingly available online in quasi-real time.

**Epidemic preparedness and response:** at the national level, there was a Public Health Emergency Operations Centre (PHEOC) handbook and a health sector emergency preparedness and response plan (HSEPRP). However, the latter was not officially approved and was not based on an up-to-date strategic risk assessment. A National PHEOC and seven Regional PHEOCs with trained staff ready to implement the IMS during emergencies were in place. However, the PHEOCs were not fully functional and lacked a legal framework, dedicated core staff, and a dedicated budget. Skilled Rapid Response Teams (RRT) were established at both the national and regional levels. Notably, the country lacked a dedicated budget line for emergency preparedness and response, relying primarily on donor and partner support. At the national level, a National Health Emergency Steering Committee was convened on an ad hoc basis for emergency response. Five of the seven regions had established Public Health Emergency Management Committees. However, none had an epidemic preparedness and response plan, and none had evaluated their preparedness and response activities within the previous year. Remarkably, 57% of the regions were familiar with the action thresholds of priority diseases. The country lacked a stockpile of emergency medicines, vaccines, and supplies, and relies on routine stocks for emergency response.

**Feedback:** the EDC produced a weekly epidemiological bulletin, but it was inconsistently published. Upon assessment, none of the facilities or regions reported receiving feedback reports regularly. Additionally, 55% of health facilities reported not receiving any feedback reports or bulletins from the central or regional levels in the previous year. In contrast, all regions indicated receiving at least two feedback reports from the national level. Furthermore, half (51%) of health facilities reported holding at least one meeting with community members within the previous six months.

### Support surveillance functions

**Availability of standard guidelines and supervision:** the country had begun the process of developing a national disease control and prevention strategy and a national disease surveillance policy. there was no disease surveillance strategic plan outlining short- and long-term surveillance goals. However, its development was anticipated to follow as a key outcome of this assessment. The third edition IDSR guidelines has not yet been printed or distributed to health facilities. Nevertheless, more than half of the health facilities visited were found to have national surveillance guidelines, primarily the IDSR Second Edition guidelines. More than 90% of health facilities reported having been supervised by higher levels at least twice over the last 6 months, while 57% of the regions reported having been supervised by the national level staff.

**Training:** at the regional level, all regional surveillance focal persons had completed a five-day IDSR training course and a three-month basic Field Epidemiology Training Program (FETP) course. Additionally, 71% of regions had focal persons who had undergone the nine-month intermediate-level training, though none had completed the two-year advanced Field Epidemiology and Laboratory Training Program. Among healthcare facilities, 88% had at least one focal person trained in IDSR, while 50% had an officer trained in basic FETP, and only 1% had personnel with intermediate FETP training.

## Discussion

This assessment provides valuable insights into The Gambia´s IDSR system´s performance, enabling the country to take stock of progress made since its adoption over two decades ago, while offering lessons for other countries. First, significant progress has been made in establishing key structures, providing normative guidance to facilitate the nationwide rollout of IDSR, and enhancing workforce capacity. However, substantial gaps persist, including the country´s exclusive use of Indicator-Based Surveillance (IBS) without the complementary implementation of Event-Based Surveillance (EBS) and Community-Based Surveillance (CBS). Additionally, weak enforcement of surveillance requirements, particularly in private facilities, continues to hinder the system´s effectiveness. The country has SCDs and action thresholds for priority diseases; however, there is a need for widespread dissemination of the updated IDSR guidelines to ensure consistent use across all surveillance sites. The presence of SCDs in most facilities is commendable, but their absence in one-third of private hospitals raises concerns about the risk of missing cases of diseases under surveillance, which could delay response to potential outbreaks. Furthermore, while access to SCDs is crucial, it should be supported by training and supervision to ensure consistent application. Even where readily available, studies in Tanzania and Nigeria have shown gaps in the actual use of SCDs [14,15].

The Gambia´s SRN was found to play a critical role in sample handling, transportation, and feedback across about 60% of health facilities with real-time tracking to improve efficiency. Its hub-and-spoke model was found to be particularly beneficial for remote areas, contributing to faster diagnoses and improved patient outcomes. However, reliance on a pre-arranged sample transportation schedule delayed case confirmation for immediately reportable diseases, while the inability to conduct international sample transport affected timely access to external reference laboratories such as the Institut Pasteur in Dakar. Furthermore, the SRN was entirely donor-dependent at the time of the assessment, hence in need of sustainable funding mechanisms.

Reporting to the regional level was a key challenge at the facility level, as a significant proportion of health facilities still relied on paper-based tools. This highlights key gaps in digital infrastructure and capacity, limiting the efficiency and timeliness of data submission. While there was considerable use of DHIS2 at regional and national levels, gaps remain in data-sharing with the laboratory, other programs, and One Health partners for coordination and integration. These findings align with the situation in many African countries that informed the second pillar of the WHO TASS flagship programme, which focuses on strengthening electronic IDSR and enhancing the use of integrated, interoperable electronic surveillance systems [9,16].

Data analysis was the poorest performing IDSR core function. While a great deal of emphasis was placed on reporting, analysis of data, coupled with the use of this data for meaningful decision-making, was limited. The low percentage of facilities and regions performing descriptive analysis weakens the system´s ability to monitor disease patterns and detect potential outbreaks early. In contrast, a marked improvement in data analysis at the facility level was reported in Sierra Leone, following the enhancement of surveillance after the Ebola outbreak [17]. In Uganda, similar improvements were attributed to the provision of IDSR training programs [18]. Utilisation of surveillance data for evidence-based decision-making is central to the effectiveness of any surveillance system and should be prioritised [19].

Significant investments have been made in developing emergency preparedness and response infrastructure, training personnel, and strengthening policy frameworks, reflecting efforts to improve emergency management. Indeed, The Gambia's diverse health workforce, with varying expertise across sectors, has enhanced the Gambian FETP and the rollout of IDSR trainings [20]. However, these investments remain hindered by a lack of official endorsement, outdated risk assessments, a lack of dedicated PHEOC core staff, and insufficient legal and financial frameworks. Additionally, the absence of regional preparedness plans, lack of evaluations for course correction, and partial familiarity with the action thresholds for priority diseases weaken outbreak management and increase the risk of delayed interventions.

Significant investments have been made in developing emergency preparedness and response infrastructure, training personnel, and strengthening policy frameworks, reflecting efforts to improve emergency management. Indeed, The Gambia's diverse health workforce, with varying expertise across sectors, has enhanced the Gambian FETP and the rollout of IDSR trainings [20]. However, these investments remain hindered by a lack of official endorsement, outdated risk assessments, a lack of dedicated PHEOC core staff, and insufficient legal and financial frameworks. Additionally, the absence of regional preparedness plans, lack of evaluations for course correction, and partial familiarity with the action thresholds for priority diseases weaken outbreak management and increase the risk of delayed interventions.

## Conclusion

This assessment has highlighted key successes and challenges within The Gambia´s surveillance system, reflecting measurable progress across the IDSR core and support functions. Notable investments in emergency preparedness and response infrastructure, workforce capacity, and sample transportation have strengthened the system´s functionality. However, gaps remain in data analysis, implementation of eIDSR, subnational laboratory capacity, and preparedness planning. Improving coordination across programs and One Health partners, securing sustainable funding mechanisms, and implementing EBS and CBS present key opportunities to enhance the system´s performance, contributing towards national and global health security. Further, key priority actions include reactivating regional emergency committees, institutionalizing multi-sectoral collaboration, ensuring consistent access to surveillance tools, and enhancing preparedness through strategic risk assessments and regional planning. Strengthening surveillance in the private sector, expanding laboratory capacity, and investing in data analytics and digital platforms will further reinforce the system´s ability to detect and respond promptly to public health threats.

### What is known about this topic

- In the WHO African region, IDSR is considered the “vehicle” for IHR implementation by providing a framework for strengthening capacities required by the IHR;
- Public health surveillance is crucial for the planning, implementation, and evaluation of public health practice, ensuring that actions are data-driven, timely, and effective in improving population health outcomes.

### What this study adds

- Provides a baseline assessment of The Gambia’s IDSR system, identifying strengths, gaps, and areas for improvement;
- Highlights critical issues that need attention, such as limited use of data for meaningful decision making and the need for digital infrastructure improvements to enhance timely detection and reporting;
- Outlines actionable recommendations, including improving coordination across surveillance programs, strengthening laboratory capacities, and expanding emergency preparedness planning to improve national and global health security.
